# Effects of norepinephrine on microglial neuroinflammation and neuropathic pain

**DOI:** 10.1002/ibra.12001

**Published:** 2021-12-08

**Authors:** He‐Lin Zou, Juan Li, Jun‐Li Zhou, Xi Yi, Song Cao

**Affiliations:** ^1^ Guizhou Key Laboratory of Anesthesia and Organ Protection Zunyi Medical University Zunyi Guizhou China; ^2^ Department of Pain Medicine Affiliated Hospital of Zunyi Medical University Zunyi Guizhou China

**Keywords:** anxiety, depression, microglia, neuroinflammation, norepinephrine, pain

## Abstract

Norepinephrine (NE) is an important neurotransmitter in the central nervous system. NE is released from locus coeruleus neurons and is involved in a variety of physiological and pathological processes. Neuroinflammation is a common manifestation of many kinds of neurological diseases. The activation of microglia directly affects the status of neuroinflammation. Several kinds of adrenergic receptors, which anchor on microglia and can be regulated by NE, affect the activation of microglia and neuroinflammation. NE influences chronic pain, anxiety, and depression by regulating the activation of microglia.

## Graphical abstract

1. Abnormally activated microglia release inflammatory mediators and cause neuroinflammation.
2. Persistent neuroinflammation may cause anxiety/depression.
3. Neuropathic pain and neuroinflammation affect each other to form a vicious circle.
4. Norepinephrine released from the locus coeruleus inhibits abnormal microglial activation and relieves chronic pain.

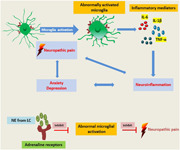

## 1 INTRODUCTION

Norepinephrine (NE) in the brain is derived from the locus coeruleus (LC).^1^, ^2^ Most areas of the brain and spinal cord are regulated by NE to maintain or enhance their wakefulness and excitability. As an important immune cell in the brain, microglia maintain the homeostasis of the central nervous system (CNS). The abnormally activated microglia release inflammatory mediators, which cause neuroinflammation, damage the nervous system, aggravate neuropathic pain, and can also play a role in the formation and maintenance of anxiety and depression. Eventually, chronic neuropathic pain, anxiety and depression mutually interact with each other, creating a vicious circle. NE acts on microglia and regulates the release of inflammatory factors such as interleukin 6 (IL‐6), interleukin 1β (IL‐1β), and tumor necrosis factor α (TNF‐α); therefore, NE regulates neuroinflammation, neuropathic pain, anxiety, and depression.

### 1.1 Abnormal activation of microglia causes neuroinflammation

Microglia are important immune cells in the CNS. They originate from the embryonic yolk sac and are distributed in the neuroepithelium. Unlike other cells in the CNS, microglia and peripheral macrophages have a common origin and have a strong regenerative ability to maintain sufficient numbers to serve their functions. Neuroinflammation has two main functions: elimination of the cause of damage and repair of damaged tissue. However, this defense mechanism may evolve into a vicious circle, resulting in continuous immune cell activation.^3^ Microglia are involved in the occurrence of a variety of neurodegenerative diseases through continuous monitoring and observation of the microenvironment of the CNS; they can sense harmful signals and respond to the insulted site, regulate neuroinflammation in the CNS, and play an important role in neurodevelopment, synaptic pruning, nerve development, apoptosis, the maintenance of synaptic plasticity, and immune monitoring.^4^ Neuroinflammation in the brain is characterized by the activation of microglia in the CNS and the release of proinflammatory factors that can cause synaptic dysfunction and neuronal death, and inhibit neurogenesis,^5^ including IL‐1β, IL‐6, and TNF‐α, chemokines (such as C–C motif chemokine ligand 1 (CCL1), CCL5 and C–X–C motif chemokine ligand 1 (CXCL1), and small‐molecule messengers (such as prostaglandins, nitric oxide [NO], and reactive oxygen species). IL‐1β causes synaptic damage by increasing the production of prostaglandin E2, leading to the release of presynaptic glutamic and postsynaptic *N*‐methyl‐d‐aspartate (NMDA) receptor activation; TNF‐α inhibits the nuclear factor‐κB (NF‐κB) pathway by activating TNF receptor 1 (TNFR1), and leads to neuron death.^5^ Studies have shown that there are two phenotypes for microglia activation: proinflammatory phenotype (M1) and anti‐inflammatory phenotype (M2). The M1 phenotype promotes the release of proinflammatory cytokines by upregulating the expression of surface molecules such as CD68 and CD16/32. In the M1 phenotype, the activation of nucleotide‐binding oligomerization domain‐like (NOD‐like) receptor pyrin‐containing 3 (NLRP3) stimulates the secretion of chemokines and cytokines,^6^ such as IL‐1, IL‐6, and TNF‐α, and other inflammatory mediators (such as inducible NO synthase, cyclooxygenase, and matrix metalloproteinases). Microglia also accumulate at the site of nerve injury to produce a more effective immune response,^7^ while the activation of M2 type microglia with anti‐inflammatory properties can inhibit the nerve damage and toxic response induced by M1 activation. Microglia induce the M2 phenotype by uptake of apoptotic cells or myelin fragments and anti‐inflammatory cytokines (IL‐4, IL‐10, and transforming growth factor), reduce neuroinflammation and neuronal damage, promote the release of neurotrophic factors, and participate in the promotion of anti‐inflammatory, tissue repair and neurogenesis,^8^ and other processes, which are conducive to the survival of neurons. However, according to recent research results, microglia M1 type activation and M2 type activation always do not exist alone in CNS. Most of the time, M1 type activation and M2 type activation coexist.^9^ For example, in Alzheimer's disease, amyloid accumulation causes small glial cell activation and neuroinflammation^10^; microglia are activated at the same time with M1 type and M2 type activation. In addition, human leukocyte antigen DR (HLA‐DR), a classic immunochemical marker of microglia, lacks specificity in identifying proinflammatory and anti‐inflammatory phenotypes.^11^ Nevertheless, this classification is still widely used, that is, microglia can be protective (M2 type activation) or noxious (M1 type activation) under different circumstances. In short, the abnormal activation of microglia regulates CNS inflammation by producing immune responses, regulating the release of proinflammatory or anti‐inflammatory factors, and participating in processes such as neuronal phagocytosis or neurogenesis.

## 2 NE REGULATES INFLAMMATORY ACTIVATION OF MICROGLIA

### 2.1 NE regulates microglia activation through adrenergic receptors

Experiments have shown that NE acts on different types of cells through adrenergic receptors belonging to the G protein‐coupled receptor family, and these receptors have different expression profiles on different cells. Microglia can be regulated by NE signals by expressing mRNA encoding α1‐, α2‐, β1‐, and β2‐adrenergic receptors.^12^ NE and β‐adrenergic agonists increase the level of cyclic adenosine monophosphate (cAMP) in microglia by activating the β2‐adrenergic receptor (β2‐AR) and inhibit proinflammatory cytokines (IL‐6, TNF‐α, and free radicals) that control the branching, monitoring, and response to damage of microglia. Coincidentally, the amount of β2‐AR expressed by microglia is higher than that of any other cell in the brain.^12^, ^13^ The NE in the brain arises from LC, and is widely transported to various areas of the brain to affect sleep, wakefulness, anxiety, and depression. Studies have indicated that damage to the LC can aggravate the activation of microglia and cause neuroinflammation. After LC degeneration, secretion of NE is reduced or absent, and the microglia controlled by NE projection are activated, the phenotype of microglia changes, and the release of IL‐6, IL‐1β, and TNF‐α and other cytokines causes neuroinflammation,^4^ suggesting that NE plays a key role in the activation of microglia. It has been reported that the levels of acetylcholine (Ach), dopamine, serotonin, and NE are reduced during general anesthesia; in addition, compared with awake mice, the microglia of anesthetized mice have an enhanced immune response to injury.^14^ Mercan et al. administered these neuromodulators to the brains of mice under isoflurane anesthesia and found that only NE can prevent anesthesia induction from affecting microglia.^13^ Cao et al. compared the microglial neuroinflammation in APP/PS1 mice and aging mice, and the degree of degenerative changes in the LC–NE system, and found that compared with aging mice, the activation of microglia in the spinal cord of APP/PS1 mice is increased, the expression of proinflammatory cytokines is upregulated, and the projection of NE neurons is reduced. In addition, the neuroinflammation of the brain and spinal cord of APP/PS1 mice increases and is accompanied by a decrease in the number of LC–NE neurons and nerve fiber projections. The large reduction in LC–NE nerve fibers may affect the release of NE transmitters in their projection areas.^15^ In wild‐type mice, pretreatment with the β‐blocker propranolol can significantly inhibit the activation of microglia in the whole‐brain, such as the hippocampus, the thalamus and hypothalamus; On the other hand, compared with wild‐type mice, β1‐AR and β2‐AR double‐gene knockout mice showed significant inhibition of stress‐induced activation of microglia in the brain, indicating that microglia interact with the NE of the whole brain through β1‐AR and β2‐AR.^16^

### 2.2 NE affects the surveillance function of microglia in the brain parenchymal function

Microglia are immune cells in the brain that interact with neurons in the brain parenchyma to continuously monitor the CNS actively and maintain the dynamic balance of the brain. In the CNS, NE acts as an endogenous signal substance to control the level of cAMP in microglia to regulate its nano‐level monitoring of brain parenchyma.^17^ Mori et al. proved that NE inhibits the proliferation of microglia by increasing the level of cAMP in microglia through β2‐AR,^12^ reducing the monitored area. Moreover, adrenergic signals were observed to control the surveillance phenotype of microglia in the awake state, and that tonic NE inhibited the surveillance of microglia protrusions in awake mice through β2‐AR. Local neuron activity affects the release of NE; drug inhibition of β2‐AR or chemical inhibition of LC release NE can lead to prolongation of microglia and increased monitoring range.^18^ Under anesthesia, of the four potential neuromodulators (Ach, dopamine, NE, and serotonin), only NE can prevent the elongation of microglia, exogenous NE can suppress the increase in monitoring of microglia protrusions caused by beard trimming and optogenetic suppression. In addition, the pharmacological inhibition of microglia β2‐ARs and the chemogenetic inhibition of LC–noradrenergic neurons increased the monitoring of microglia protrusions in awake mice.^18^ It is worth noting that NE also reduces the contact area between microglia and neuron dendrites and their direct contact time, thereby reducing the neuronal damage.^13^ This evidence indicates that the real‐time monitoring of CNS by microglia is regulated by NE. When NE secretion is abnormal, the monitoring of CNS by microglia will be affected, the contact time of microglia protuberances and dendrites will be prolonged, the contact area will increase, the interaction between microglia and neurons is enhanced, and the neurons are damaged.

### 2.3 Microglia, the noradrenergic system, and neuropathic pain

It is now generally believed that neuropathic pain is related to the central sensitization of the spinal cord and brain.^19^ The activation and proliferation of microglia and the changes in the expression of neuroinflammatory factors caused by transcription and posttranscriptional regulation play an important role in the generation and maintenance of central sensitization and neuropathic pain.^20^ The activation state of microglia is regulated by NE, suggesting that the occurrence and development of neuropathic pain are regulated by NE too. In addition, the LC–spinal cord noradrenergic descending pathway is important to regulate microglia activation and neuropathic pain, and is also a potential target for pain treatment.^21^

### 2.4 Microglial neuroinflammation and neuropathic pain

During the transition from acute pain to chronic pain, peripheral injury and excessive activity of primary sensory neurons release proinflammatory cytokines (such as TNF‐α and IL‐1β), chemokines, glutamate, and reactive oxygen species through activated astrocytes and microglia to promote neuroinflammation. As the headquarters of the nervous system, the brain has an impact on the occurrence and development of neuropathic pain. In the process of pain recognition, multiple brain regions were activated at the same time, while the activation of microglia in the prefrontal cortex (PFC), the anterior cingulate cortex (ACC),^22^ and the hippocampus^21^, ^23^ may be responsible for persistent neuropathic pain. The activated microglia promote the release of proinflammatory cytokines, induce neuroinflammation, and enhance long‐term noxious nerve signal transmission, leading to central pain sensitization, causing and aggravating neuropathic pain.^20^ After peripheral nerve injury (PNI), TNF‐α can differentially regulate hippocampus and spinal cord synaptic plasticity through a microglia‐dependent mechanism.^23^ Blocking the TNF‐α signaling pathway and the IL‐1β signaling pathway can alleviate the pain hypersensitivity of neuropathic pain in rodents.^24^ The activation of microglia in the spinal dorsal horn to secrete a large number of proinflammatory factors also plays an important role in the occurrence and development of neuropathic pain. Brifault et al. found that in mice lacking the gene encoding the plasma membrane receptor LDL receptor‐related protein‐1 (LRP1) in microglia after partial sciatic nerve ligation (PNL), the lack of LRP1 in microglia prevents the development of tactile hypersensitivity and also significantly reduces the activation of spinal dorsal horn microglia and the expression of proinflammatory cytokines after PNL, indicating that the activation of spinal cord microglia and the development of neuroinflammation in the spinal dorsal horn are related to the occurrence of neuropathic pain‐related behaviors. A single gene deletion in microglia is sufficient to prevent the occurrence of analgesia caused by trigeminal ganglion injury.^25^ The G‐protein‐coupled receptor 34 gene (GPR34) is considered to be a gene present in microglia. Sayo et al. found that in mice lacking GPR34, GPR34‐mediated signal expression deletion inhibits the proinflammatory response of microglia and reduces the neuropathic pain caused by nerve injury.^26^ In chronic or sustained stress load mouse models, ATF3, a marker of neuronal damage or overactivation, was first expressed in lumbar dorsal root ganglion (DRG) neurons 2 days after sustained stress was initiated; on the fifth and sixth days, a large number of microglia gathered around the subgroups of motor neurons in the inner and ventral dorsal horns of the spinal cord, and the motor neurons surrounded by microglia were positive for ATF3 and projected mainly to the soleus muscle. The electromyographic activity of the soleus muscle in the sustained stress group was two to three times that in the control group, which proved that the activation of microglia induced by chronic proprioceptors may play a key role in the initiation and maintenance of abnormal pain in patients with chronic fatigue syndrome, and the duration of pain is related to the degree of microglia activation.^27^ Ma et al. found that inhibiting neuroinflammation caused by the activation of microglia in the spinal dorsal horn can reduce neuropathic pain caused by herpes zoster in rats. They believe that neuropathic pain in the rat spinal dorsal horn and microglia in the brain are activated when different glial transmitters are released at different times, all of which have an impact on the occurrence and development of neuropathic pain.^20^ In short, the results of the research on the expression of related genes in microglia and the related behavioral research results after inhibiting the activation of microglia all illustrate the fact that elevated microglial neuroinflammation is involved in the occurrence and development of neuropathic pain. Inhibiting the inflammatory activation of microglia or the expression of related genes during the activation of microglia could be a new way to treat neuropathic pain.

### 2.5 LC–noradrenergic system affects the occurrence and development of chronic pain

In animal models of chronic pain, different types of pain events have been studied in depth at the molecular and cellular levels, and it has been recognized that in the transition from acute pain to chronic pain, the noradrenergic LC–spinal cord descending pathway inhibits the transmission of pain. The descending pain inhibition pathway (large multipolar neurons) originating from the ventral side of the LC projects to the spinal dorsal horn, reducing the transmission of spinal pain and plays a prominent role in endogenous analgesia.^28^ It is believed that the analgesic gabapentin compounds activate the noradrenergic descending pathway from the LC to the dorsal horn of the spinal cord by inhibiting the release of γ‐aminobutyric acid (GABA) and inducing the release of glutamate, and increasing the NE level in the spinal cord to achieve an analgesic effect.^29^ In addition, under the pathological conditions of pain, stimulation of histamine H4 receptors in LC activates the descending pain inhibition pathways, inhibits the excitement of spinal dorsal horn neurons, and achieves an analgesic effect.^30^ After that, animal studies have shown that in the relatively early stage of chronic pain, the noradrenergic descending pathway can effectively inhibit mechanical pain and thermal hypersensitivity by increasing the level of brain‐derived neurotrophic factor (BDNF).^21^ The antinoxious effects of NE in the dorsal horn of the spinal cord involve multiple mechanisms. NE stimulates the coupling of presynaptic and postsynaptic α2‐AR with inhibitory G protein (Gi/o) and stimulates the synapses on primary nociceptive neurons, and pre‐α2‐AR inhibits voltage‐gated calcium channels, thereby reducing the release and transmission of noxious neurotransmitters (glutamate and substance P).^31^ On the other hand, stimulating the postsynaptic α2‐AR activates the secondary sensory neurons of the spinal cord, which leads to hyperpolarization by opening the inflow potassium channels, thereby reducing the excitability of the neurons.^32^ Sanna et al. found that an intrathecal injection of the α2‐AR agonist clonidine can reduce mechanical and thermal hyperalgesia caused by sciatic nerve injury (SNI).^30^ Impaired endogenous adrenergic analgesia is believed to be the cause of the transition from acute pain to chronic pain.^21^ In fact, in the later stage of chronic pain, the NE neurons in the LC become weaker in response to noxious stimuli, which is due to the dysfunction of the glutamatergic system that controls the release of NE; the defect in the function of the noradrenergic descending pathway promoted the transition from acute pain to chronic pain in animal models.^33^ These findings suggest a possible strategy to alleviate chronic pain by restoring the function of the noradrenergic LC–spinal cord descending pathway.

### 2.6 NE uptake inhibitors relieve neuropathic pain by inhibiting microglia activation

Tricyclic antidepressants (TCAs) and serotonin norepinephrine reuptake inhibitors (SNRIs) are recommended as first‐line drugs for the treatment of various neuropathic pains. Zhang et al. discussed the mechanism of amoxetine, one of the NE reuptake inhibitors, and observed the activation of microglia and astrocytes, the protein levels of proinflammatory cytokines, and the expression of mitogen‐activated protein kinase and NF‐κB. They observed the direct effect of amoxetine on the activation of microglia and its signal transduction mechanism, and found that after 4 weeks of treatment with amoxetine, the pain sensation of rats with neuropathic pain was significantly reduced. Amoxetine also reduced the activation of rat spinal cord microglia, the accumulation of proinflammatory cytokines, and the activation of p38 and c‐jun amino‐terminal kinase (c‐JNK).^34^ Duloxetine, another NE reuptake inhibitor, has a significant inhibitory effect on the function of the microglia purinergic receptor P2X4R (a subtype of ATP‐gated nonselective ion channels). In the CNS, microglia mainly express P2X4R, which is highly upregulated in spinal microglia after PNI. Blocking the function of P2X4R can reverse mechanical atopic pain. In addition, duloxetine significantly reduced the increase in Ca^2+^ levels induced by ATP, that is, inhibiting the activity of P2X4R in microglia.^35^ These studies show that the NE reuptake inhibitors relieve the pain by inhibiting the inflammatory activation of microglia and microglial neuroinflammation.

## 3 MICROGLIA, LC–NORADRENERGIC SYSTEM AND ANXIETY/DEPRESSION

### 3.1 Microglia activation may cause anxiety and depression

Recent studies have shown that depression is accompanied by neuroinflammation, especially characterized by the activation of microglia; depression is closely linked to abnormal microglia function.^36^ Rats that were subjected to chronic mild stress (CMS) for 12 weeks showed obvious depression and anxiety‐like behaviors; in addition, the activation of microglia in the hippocampus was detected, NLRP3 was also activated, and the expressions of IL‐1β, IL‐6, and other inflammatory mediators were upregulated, suggesting that neuroinflammation induced by microglia activation may play an important role in the pathogenesis of CMS‐induced depression‐like behavior.^37^ Continuous activation of microglia in the brains of mice with spinal cord injury was observed; however, after depletion of microglia with colony‐stimulating factor 1 receptor (CSF1R) antagonist PLX5622, the PLX5622 treatment caused a decrease in microglia in the brain and spinal cord, and the recovery of neuroinflammation in the brain and damaged spinal cord, the cognitive, depression‐like behavior, and motor function of mice also gradually improved.^38^ Furthermore, IL‐4 drives the phenotypic transition of microglia in the hippocampus to mediate the neuroprotective effect of the CNS, promotes the growth of nerves dependent on BDNF, and improves depression‐like symptoms.^39^ In short, for a long time, microglia cause neuroinflammation and neurodegeneration, increase neuronal oxidative irritability, and mediate anxiety‐ and depression‐like behavior. Reducing the number of microglia in the relevant area or inhibiting the activation of microglia can improve the degree of neuroinflammation and control the symptoms of anxiety and depression.

### 3.2 Chronic pain is accompanied by microglia activation and anxiety/depression

It is now generally believed that neuroinflammation caused by microglia activation in neuropathic pain is related to comorbidities such as anxiety and depression.^40^ In rats with SNI, neuropathic pain and depressive behavior coexist, and the activated microglia and inflammatory factors increase at PFC, indicating that the activation of microglia is closely related to neuropathic pain and depression.^37^ The rats showed chronic mechanical hypersensitivity after chronic constriction injury (CCI); at the delayed time point (more than 1 month) after the onset of pain, microglia at PFC and hippocampus were activated and the expression of TNF‐α was upregulated; depression‐like behavior was observed 8 weeks after CCI. The authors believe that microglia in specific brain regions were activated at a delayed time point and played a role in the development of related affective disorders in the mice.^40^ More evidence of functional magnetic resonance imaging studies in patients with postherpetic neuralgia (PHN) suggests that there are abnormal functional activities in the regions of the brain linked to anxiety and depression.^41^ Most PHN patients also have anxiety and depression that may be related to the activation of microglia at the PFC, ACC, and hippocampus.^20^ Negrete et al. injected intra‐articular sodium iodoacetate in kappa opioid receptor knockout mice, predynorphin knockout mice, and wild‐type mice to induce osteoarthritis; the results showed that all mice had abnormal pain, and the activation of microglia in the spinal cord and lumbar showed a similar increase. At the same time, the mice also showed obvious anxiety depression‐like manifestations.^42^ In chronic neuropathic pain, the activation of microglia in the nervous system causes neuroinflammation, which affects the occurrence and development of anxiety and depression symptoms.

### 3.3 LC–noradrenergic system intervention relieves anxiety and depression caused by microglial inflammation

In the treatment of anxiety and depression, noradrenergic system intervention may play an important role. Several recent studies support the theoretical hypothesis that increasing cholinergic signals through NE tension can cause anxiety and depression, which suggests that increased cholinergic signals can lead to symptoms of depression, and NE signals can act on the sympathetic nervous system and specific brain regions to regulate the cholinergic signals and exert anti‐anxiety and antidepression effects, which indicates that intervention in the noradrenergic system may relieve anxiety and depression.^43^ In mice with long‐term cognitive impairment and depression, after treatment with mirtazapine for 21 days, metabolomics studies showed that mirtazapine increased the production of NE and decreased the inflammatory activation of microglia, and it also alleviated the symptoms of depression in mice.^44^ Serikuly et al. confirmed that arecoline has anti‐anxiety effects on zebrafish, and can also lead to the development of social preferences in mice, increase the NE levels in the brain, the increase the expression of microglia‐specific genes egr2 and ym1 in the brain.^45^ Interestingly, these two genes represent the activated M2 microglia subtype (which plays a protective role in the brain), rather than the M1 microglia subtype, which triggers neuroinflammation.^43^ The increase in the NE level in the brain induces the activation of the M2 type of microglia and exerts an anti‐anxiety effect. The α2‐AR agonist guanfacine is an anti‐anxiety and depression drug, and it is reported that the key to guanfacine in the treatment of anxiety and depression lies in activating α2‐AR in the amygdala. α2‐AR has both presynaptic and postsynaptic distributions, and low concentrations of NE can activate α2‐AR,^43^ inhibit microglia activation, reduce neuroinflammation, and improve cognitive function. SNRIs, as a first‐line drug for the treatment of generalized anxiety disorder and social anxiety disorder, inhibit the inflammatory activation of microglia by increasing the levels of NE in the CNS and reduce neuroinflammation. While treating chronic pain, it also relieves the symptoms of anxiety and depression. Increased amygdala responsiveness is a characteristic pathological manifestation of patients with anxiety. A large number of animal and clinical studies have shown that LC regulates the amygdala responsiveness through NE projection, which in turn affects the occurrence and development of anxiety and depression.^46^ Therefore, intervention of the noradrenergic system may be an important direction for studying the occurrence and treatment of anxiety and depression.

## 4 CONCLUSION AND PROSPECTS

**Figure 1:**
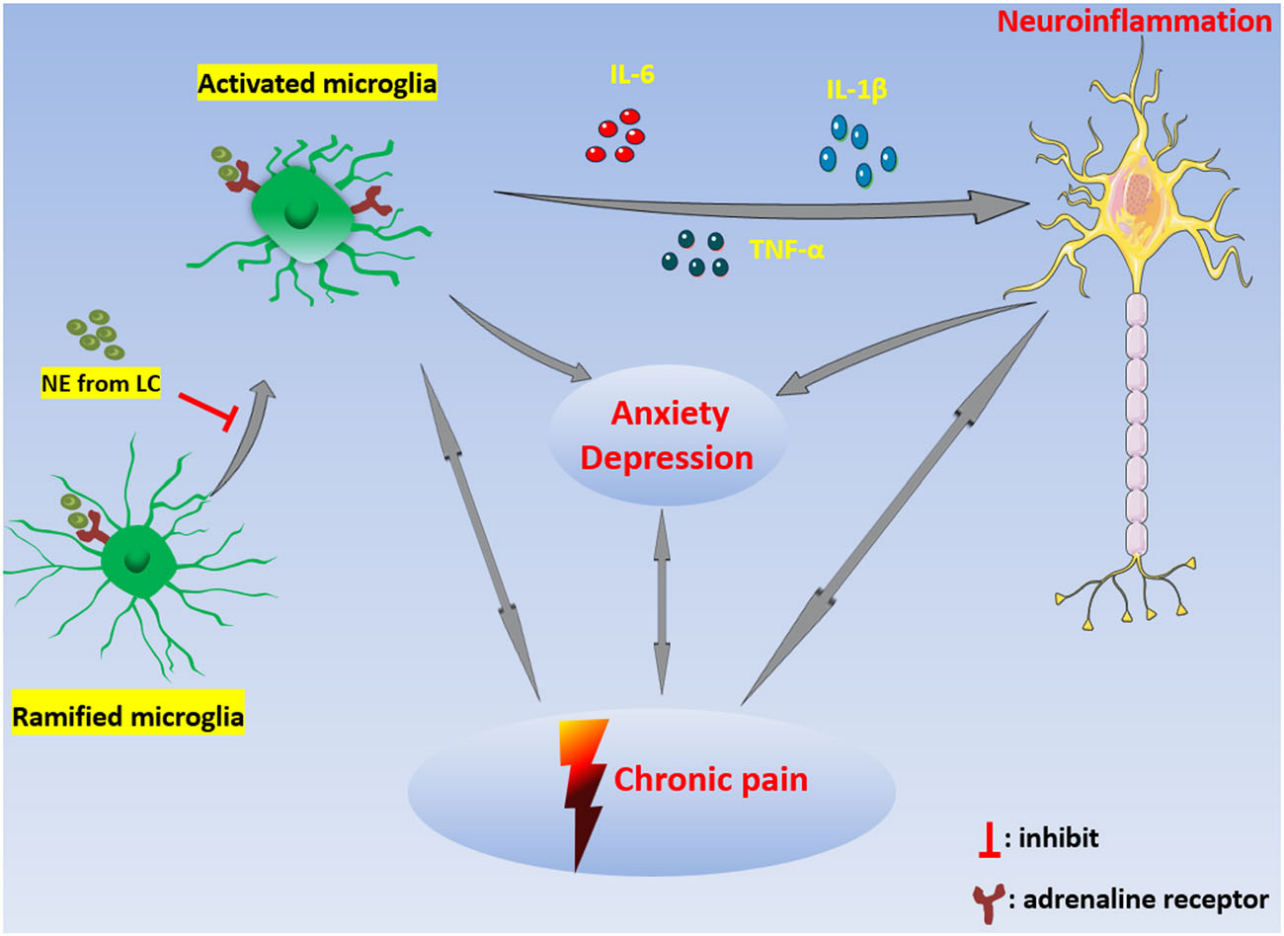
Interaction between microglial activation, neuroinflammation, neuropathic pain, anxiety, and depression. The abnormal activation of microglia releases inflammatory mediators and causes neuroinflammation. Continuous neuroinflammation aggravates neuropathic pain, anxiety, and depression. Neuroinflammation, neuropathic pain, and anxiety/depression promote each other to form a vicious circle. LC, locus coeruleus; NE, noradrenaline/norepinephrine [Color figure can be viewed at wileyonlinelibrary.com]

The activation of microglia is closely related to neuroinflammation. Continuous neuroinflammation aggravates neuropathic pain and may lead to anxiety and depression. In addition, anxiety, depression, and chronic pain mutually aggravate each other and form a vicious circle (Figure 1), which makes treatment of pain difficult. As a regulator of microglia activation, NE affects pain, anxiety, and depression by regulating neuronal excitability and neuroinflammation. Further studies are needed to clarify the specific mechanism of NE on microglia neuroinflammation, to explore whether NE can regulate the activation state of microglia, and to find possible drug targets to develop effective treatments for neuropathic pain, anxiety, and depression.

## CONFLICT OF INTERESTS

The authors declare that there are no conflict of interests.

## ETHICAL STATEMENT

There are no possible animal or medical ethical issues for this review article.

## AUTHOR CONTRIBUTIONS

He‐Lin Zou and Song Cao contributed to the main conception of this review that led to the submission and resources collecting; He‐Lin Zou, Jun‐Li Zhou, Xi Yi, and Juan Li contributed to the draft and later editing; and Song Cao finalized the review and approved the final version.

## Data Availability

The data reported in this study are available from the Lead Contact on request.
